# Efficacy of NSAID Transdermal Patch for Postoperative Management in Total Knee Arthroplasty

**DOI:** 10.3390/jcm14228098

**Published:** 2025-11-15

**Authors:** Khanawan Tubsrinuan, Paphon Sa-ngasoongsong, Chavarat Jarungvittayakon, Kulapat Chulsomlee, Siwadol Wongsak

**Affiliations:** 1 Department of Orthopedics, Faculty of Medicine Ramathibodi Hospital, Mahidol University, Bangkok 10400, Thailand; t.kanawan@gmail.com (K.T.); paphon.sag@mahidol.ac.th (P.S.-n.); chavarat.jar@mahidol.ac.th (C.J.); drkulapat.chu@gmail.com (K.C.); 2 Chakri Naruebodindra Medical Institute, Faculty of Medicine Ramathibodi Hospital, Mahidol University, Samut Prakan 10540, Thailand

**Keywords:** OA knee, pain control, dexamethasone, multimodal analgesia, KOOS, VAS

## Abstract

**Background:** While topical NSAID patches are effective in non-surgical knee conditions, their efficacy following total knee arthroplasty (TKA) remains understudied. The intention behind this study was the comparison of pain and functional score between esflurbiprofen and placebo patches for postoperative pain control after TKA. **Methods:** A triple-blinded randomized controlled trial was conducted among patients aged 55–80 years with primary knee osteoarthritis. Patients who had an allergy to study medications, had chronic kidney disease, had diabetes, used steroids, were unsuitable for spinal and subsartorial block, or were unwilling to participate were excluded. All eligible participants were randomized and assigned to either esflurbiprofen 40 mg transdermal patch or placebo patch starting from postoperative day 3 to 16 blindly and followed up for 3 months. Outcome assessment was a visual analog scale (VAS), morphine use, minimum daily VAS, time to minimum VAS, knee osteoarthritis outcome score, joint replacement (KOOS, JR), knee circumference, flexion angle, and adverse events. **Results:** Seventy patients underwent primary TKA (*n* = 35 each group). The average age and body mass index were 67.5 ± 13.7 years and 26.8 ± 4.5 kg/m^2^. There was no statistically significant difference in baseline characteristics between the two groups. When compared with the placebo group, the esflurbiprofen group presented statistically significantly diminished VAS scores after day 7 and morphine use at day 3 (*p* < 0.001), as well as subsequently enhanced KOOS scores at 6 weeks and 3 months (*p* < 0.001). No statistically significant difference between groups was found in terms of knee circumference, flexion angle, or time to minimum VAS (*p* > 0.05 for all). One patient in the esflurbiprofen group (2.86%) reported a mild skin reaction. **Conclusions:** Esflurbiprofen patches significantly reduce postoperative pain and improve short-term function after TKA without major complications.

## 1. Introduction

Total knee arthroplasty (TKA) is a common surgical procedure for elderly patients with end-stage osteoarthritis (OA) to alleviate joint pain and improve knee function [1]. However, TKA often results in moderate-to-severe postoperative pain with chronic postsurgical pain requiring effective postoperative pain management during the perioperative period and after discharge for patient recovery, facilitating early rehabilitation, and improving the overall outcomes [1,2,3]. The current optimal approach for TKA is multimodal analgesia, which includes preoperative, intraoperative, and postoperative analgesic regimens, to maximize the efficacy through several analgesic regimens while minimizing their adverse effects [1]. At present, non-steroidal anti-inflammatory drugs (NSAIDs) are the common medications after discharge, and their efficacy has been proven [4,5,6]. However, safety concerns need to be considered due to the association with many complications, such as acute kidney injury and gastrointestinal disturbance [7]—especially when used in combination with aspirin [8].

Topical NSAIDs patch application is one of the effective treatments for many orthopedic conditions—such as acute impact injury [9], myalgia [10], and knee osteoarthritis [11,12]—with a better safety profile than the oral NSAIDs medications [13,14]. Recently, a few studies included demonstrations of NSAIDs patch use for the perioperative management in orthopedic surgery [15,16,17], resulting in a statistically significant reduction in the perioperative pain control in the early postoperative period, especially in the first postoperative 72 h. To the best of our knowledge, there was only one study in which a topical NSAIDs patch was used after TKA. Although the previous study could demonstrate a statistically significant reduction in postoperative pain in the first and third postoperative day, several limitations had to considered as small sample size with high dropout rate (as 18.2%) in control group due to uncontrollable pain from analgesic protocol and having only a short-term (2 weeks) follow-up period without the data related to the postoperative knee functional recovery [15]. Moreover, the application of topical NSAIDs patches in previous studies varied in dosage from 1 to 2 patches per day and duration from 3 days to 2 weeks, resulting in a non-standardized protocol and different outcomes of their effectiveness [15,16,17]. Thus, the efficacy of the topical NSAIDs patch after TKA within a standardized perioperative pain management, including multimodal analgesia, remains unclear, particularly on their efficacy beyond the postoperative 2 weeks and their role in enhancing knee functional outcome.

The intention behind this study was the evaluation of the efficacy of the transdermal esflurbiprofen patch in controlling postoperative inflammation, pain, and functional recovery following TKA. We hypothesized that patients who underwent primary TKA and received a topical NSAIDs patch for 14 days would result in better postoperative pain control and knee functional recovery compared to those with a placebo control. Therefore, our objectives were to evaluate the effect of (1) esflurbiprofen patch application after operation for 14 days on postoperative pain control compared to placebo in the patients underwent primary TKA and (2) esflurbiprofen patch application after TKA on the morphine consumption, minimum VAS during a day, time to minimum VAS after applying the patch, postoperative knee osteoarthritis outcome score, joint replacement (KOOS, JR), knee circumference and knee flexion angle.

## 2. Materials and Methods

**Figure 1:**
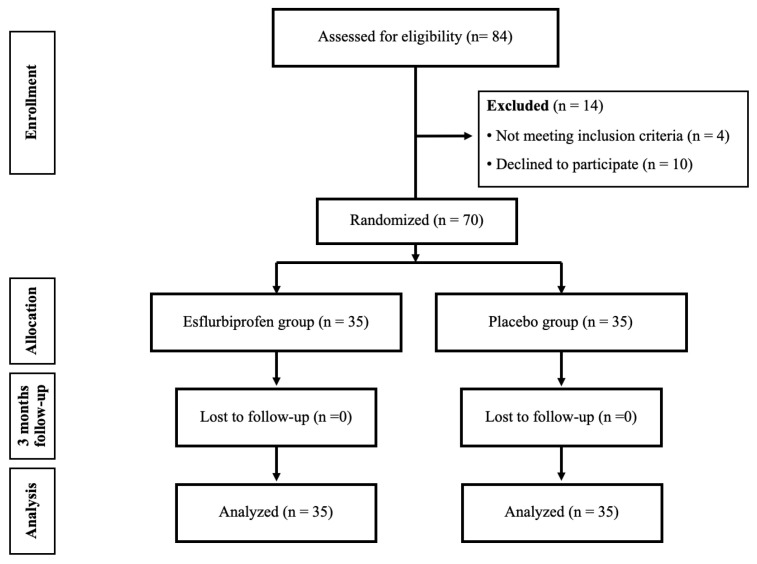
The flow chart of this study.

This study comprised a triple-blinded randomized controlled trial conducted in a medical university hospital. Prior approval was obtained from the Institutional Review Board (MURA2023/387, approved on 10 May 2023), and the trial was registered with thaiclinicaltrials.org (TCTR20230907003, approved on 7 September 2023). The study population comprised all patients who were diagnosed as primary knee OA based on 2016 revised criteria of American College of Rheumatology [18] and were scheduled for primary TKA from June 2023 and May 2024 (Figure 1). The preparatory activities started in June 2023, but the first patient enrollment was on 18 September 2023 which all patient enrollment occurred after trial registration. The inclusion criteria were as follows: (1) patients who were diagnosed as primary knee OA with a Kellgren and Lawrence [14] classification grade of 3 or 4, (2) age between 55 and 80 years, and (3) willing to participate in the study and give their informed consent. The exclusion criteria included the following: (1) history of allergy to medication used in this study including steroids, NSAIDs, morphine, paracetamol, gabapentinoids, or codeine; (2) chronic kidney disease with a glomerular filtration rate < 30 mL/min/1.73m^2^; (3) diabetes with preoperative HbA1c > 8%; (4) long-term or chronic steroid use (more than 1 month); (5) inability to undergo spinal block combined with subsartorial block; and (6) refusal to participate or withdrawal from the study.

*TKA perioperative protocol:* After patients were enrolled in the study, a standardized perioperative protocol would be applied. All patients received a standard spinal anesthesia with an additional subsartorial block. Preoperative intravenous 10 mg dexamethasone was given. All surgical procedures were performed by one of three orthopedic surgeons who have experience in hip and knee replacement for more than 10 years. All surgeons and anesthesiologists were blinded to treatment allocation and had no access to randomization or patch handling. A midline incision and medial parapatellar arthrotomy approach under pneumatic tourniquet was used. A 50 mL periarticular cocktail solution containing 0.5% bupivacaine 20 mL, ketorolac 30 mg, and cefuroxime 750 mg was injected before prosthesis insertion. The implants used in this study are either NexGen (Zimmer Biomet, Warsaw, IN, USA), ATTUNE (DePuy Synthes, Warsaw, IN, USA), or PFC SIGMA (DePuy, Synthes, Warsaw, IN, USA) implants. All prostheses were fixed with full cementation technique using bone cement with gentamicin (PALACOS^®^ R+G, Heraeus Medical GmbH, Wehrheim, Germany). The drain tube was inserted inside the joint before capsule and skin closure. Then the intraarticular tranexamic acid (IA-TXA) application using a 25 mL solution containing 500 mg tranexamic acid was injected through with drain and clamping for 2 h [19]. Compressive dressing was applied for 48 h.

After operation, all patients were treated with the same postoperative protocol. The patients were asked to ambulate as soon as possible from bedside training to full weight bearing with walking aids within 48 h. On the postoperative day 2 (POD2), drain and compressive dressing were removed, and a standard surgical dressing was applied. All patients were routinely discharged from the hospital after the postoperative day 3 (POD3).

The same perioperative and postoperative pain management was applied in this study. All patients received oral regular acetaminophen 500 mg every 6 h until they were discharged. In-hospital rescue medication was intravenous morphine 3 mg every 4 h if 10-point visual analog scale (VAS) ≥ 4. After discharge, all patients were given the same set of pain medication and instruction, including oral acetaminophen 500 mg every 6 h (30 tablets), gabapentin 300 mg as a single bedtime dose, paracetamol and codeine (300 mg/15 mg) 1 tablets every 6 h if as needed for pain with moderate intensity (VAS between 4 and 7) (20 tablets), and chlorpheniramine 4 mg every 6 h if allergic reaction occurred (5 tablets). All patients received logbook before discharge for recording daily patch changes, pill count, postoperative minimum VAS, and adverse effects. Adherence was cross-checked by telemedicine on POD7 and in-person clinic visit at POW2.

Postoperative rehabilitation was designed with home-based setting, including 20-min cold compression on the operated knee 4 times a day, 20-min sessions of progressive range-of-motion and quadriceps strengthening exercises 3 times a day for 3 months. After discharge, all patients were followed up with via phone interview on POD7 for VAS assessment and outpatient clinic visit at postoperative 2 weeks (POW2), 6 weeks (POW6), and 3 months (POM3) for further assessment.

*Randomization and study intervention:* All Patients were randomized into 2 groups (esflurbiprofen group or placebo group) using computer-generated blocked randomization with various block sizes through STATA version 17.0 (StataCorp, College Station, TX, USA). The randomization was then concealed in the sequentially numbered, opaque, sealed envelopes. The envelopes containing treatment assignments were opened sequentially by a research assistant who was not involved in the concealment process on the morning of POD3. This patch application on POD3 was chosen due to the use of drain and compressive dressing during the first 48 h. Moreover, in our experience, the duration of analgesic effect of combined spinal anesthesia and subsartorial block, intravenous dexamethasone, and periarticular injection would be able to control the postoperative pain for 48 h.

The intervention and placebo groups received daily application of a transdermal patch for 14 days as follows. The patches were applied at 8:00 a.m. each day and removed at 8:00 a.m. on the next day.

**Figure 2:**
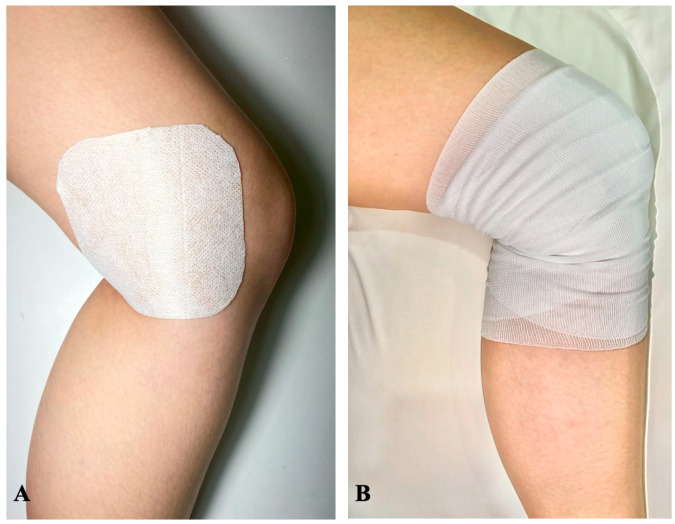
The application of transdermal patch in this study. (**A**) The patch was daily applied on the posteromedial aspect of the operated knee, that located 7–8 cm medial to the surgical wound for 14 days, from the POD3 to POW2. (**B**) After the patch application, the conforming bandage was applied to the knee to prevent the patch from dislodging.

Intervention group: the patients would receive daily application of an esflurbiprofen 40 mg transdermal patch (LOCOA^®^, TOKUHON Corporation, Saitama, Japan) and covered with an adhesive tape (Fixomull^®^ stretch, BSN medical GmbH, Hamburg, Germany) on the posteromedial aspect of the knee, 7–8 cm from the surgical site. The patch was then covered with conform bandaging for prevention of early dislodging patch. All patients were given 14 patches for 14-day period application (Figure 2A,B).

Placebo group: the patients would receive daily application of a DuoDERM^®^ dressing (Convatec Inc., Bridgewater, NJ, USA) with 0.5 mL of liquid camphor to impart a scent similar to menthol, the same as esflurbiprofen patch, and covered with the same size of adhesive tape on the same area of the knee as the intervention group and covered with conform bandaging. All patients were given 14 patches for 14-day period of application.

*Study assessment:* Demographic data as age, gender, body mass index (BMI), comorbidities, and implant used, were collected preoperatively.

The primary outcome was pain score, measured by a 10-point VAS, during the resting period (rVAS) and during the movement (mVAS). A 0-point implied no pain, while a 10-point indicated the worst pain imaginable. mVAS after TKA was defined as VAS that the patients experienced during the knee exercise on postoperative rehabilitation protocol.

The pain score was recorded preoperatively and after surgery, scheduled on 8 a.m., on POD1, POD2, POD3, POD7, POW2, POW6, and POM3.

The secondary outcomes were assessed as follows:

- The amount of morphine consumption per day was measured as the total amount of daily morphine used from POD1 to POD3.
- Postoperative minimum VAS (min-VAS) and time to min-VAS: min-VAS is defined as the lowest pain score during the day. Time to min-VAS was defined as the duration from 8 am (the time of patch application) to the time experienced of min-VAS. Both outcomes were measured on the POD4, POD5, POD6, and POD7.
- Knee circumference and knee flexion angle: Knee circumference was measured in cm at the level of 1 cm above the patella in supine position. Knee flexion angle was measured with a long-arm goniometer during active knee flexion. Both outcomes were measured preoperatively and postoperatively on the POD2 and POD3, POW2, POW6, and POM3.
- KOOS, JR was measured preoperatively and on POW2, POW6, and POM3.
- Adverse effects during the patch application were defined as application site skin complications (such as dermatitis, erythema, or eczema) and systemic adverse effects (such as anaphylaxis, gastrointestinal bleeding, or acute renal failure) during the 14-day period of use.

*Statistical Analysis:* Data were analyzed using STATA version 17, Statacorp, College Station, TX, USA. Continuous parametric variables were presented as mean and standard deviation, and non-parametric variables were presented as median and interquartile range. The difference between two groups was compared via Student’s *t*-test (parametric) or Mann–Whitney U test (non-parametric), while overall comparison from multiple episodic recorded data was analyzed via repeated measures ANOVA method. A chi-square or Fisher’s exact test was used to compare binary or categorical data. *p*-value < 0.05 was considered statistically significant.

*Sample Size Calculation:* The sample size estimation was calculated from the data from pilot study in 20 patients who underwent primary TKA in our hospital, using the VAS score on motion on POD7. The mean ± SD of 10 mm VAS was 4.5 ± 2.4 mm. The following parameters, as alpha error (α) = 0.05, power of the study (1 − ß) = 0.8, difference in population means (δ) as minimally clinical important difference = 1.8 mm [20], and ratio between groups = 1:1, were applied. Therefore, the sample size required for each group was 28 participants. After adding with 25% dropout rate (*n* = 7), the final sample size was a total of 70 participants (35 per group).

## 3. Results

### 3.1. Demographic Data

Table 1 shows the demographic data of this study. The average age and BMI in this study were 67.5 ± 13.7 years and 26.8 ± 4.5 kg/m^2^. Most of these patients were female (63.0%). There was no statistically significant difference in patients’ characteristic data between the esflurbiprofen and placebo groups (*p* > 0.05 for all). None of the TKA-related complications, including periprosthetic fracture, infection, and deep vein thrombosis, was found in this study.

### 3.2. Pain Outcomes

**Figure 3:**
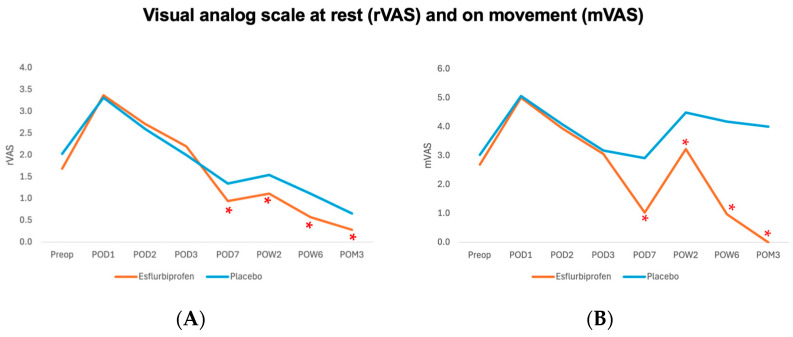
Visual analog scale at rest (rVAS) (**A**), and on movement (mVAS) (**B**) outcome. Orange and blue lines represent Esflurbiprofen and placebo group, respectively. (* = statistically significance with *p* < 0.05).

- *VAS at rest (rVAS) and VAS on movement (mVAS)*. Before the patch application, both groups showed a non-significant difference in resting rVAS and mVAS from the preoperative period to POD3 (*p* > 0.05 for all). However, after the patch application, the esflurbiprofen group demonstrated a statistically significant reduction in both rVAS and mVAS from POD7 to POM3 (*p* < 0.05 for all) (Table 2 and Figure 3).

**Table 2:** Visual analog scale (VAS) primary outcomes as VAS at rest (rVAS) and VAS on movement (mVAS).

	VAS at Rest (rVAS)				VAS on Movement (mVAS)			
	Esflurbiprofen Group	Placebo Group	*p*-Value	Δ [95% CI]	Esflurbiprofen Group	Placebo Group	*p*-Value	Δ [95% CI]
	(*n* = 35)	(*n* = 35)			(*n* = 35)	(*n* = 35)		
Preop	1.7 ± 1.4	2.0 ± 1.1	0.24	−0.3 [−0.9, 0.2]	2.2 ± 1.6	2.0 ± 1.3	0.47	0.2 [−0.4, 1.0]
POD1	3.4 ± 1.3	3.3 ± 1.2	0.85	0.1 [−0.5, 0.6]	5.0 ± 1.6	5.1 ± 1.4	0.87	−0.1 [−0.8, 0.6]
POD2	2.7 ± 1.1	2.6 ± 0.7	0.61	0.1 [−0.3, 0.5]	3.9 ± 1.2	4.1 ± 1.2	0.61	−0.2 [−0.7, 0.4]
POD3	2.2 ± 1.1	2.0 ± 0.9	0.41	0.2 [−0.3, 0.7]	3.1 ± 1.0	3.2 ± 0.9	0.63	−0.1 [−0.6, 0.4]
POD7	0.9 ± 0.9	1.3 ± 0.6	0.03 *	−0.4 [−0.8, −0.0]	1.0 ± 0.7	2.9 ± 0.7	<0.001 *	−1.9 [−2.2, −1.6]
POW2	1.1 ± 0.7	1.5 ± 0.7	0.01 *	−0.4 [−0.8, −0.1]	3.2 ± 1.4	4.5 ± 1.1	<0.001 *	−1.3 [−1.8, −0.7]
POW6	0.6 ± 0.6	1.1 ± 0.7	0.002 *	−0.5 [−0.9, −0.2]	1.0 ± 1.5	4.2 ± 1.0	<0.001 *	−3.2 [−3.8, −2.6]
POM3	0.3 ± 0.5	0.7 ± 0.7	0.01 *	−0.4 [−0.7, −0.1]	0.0 ± 0.0	4.0 ± 1.0	<0.001 *	−4.0 [−4.3, −3.7]

POD: postoperative day, POW: postoperative week, POM: postoperative month. Δ; mean difference between the esflurbiprofen group and placebo group, CI; confidence interval. *; significant difference with *p* < 0.05.

- *Morphine consumption.* During POD1 and POD2, there was no significant difference in morphine consumption between the groups. However, the esflurbiprofen group showed a statistically significant reduction in morphine consumption compared to the placebo group on POD3 (0.1 ± 0.5 mg versus 2.6 ± 1.8 mg, *p* < 0.001) (Table 3).

**Table 3:** Comparison of the postoperative use of morphine consumption (mg).

	Esflurbiprofen Group	Placebo Group	*p*-Value
	(*n* = 35)	(*n* = 35)	
POD1	3.5 ± 1.1	5.4 ± 2.6	0.62
POD2	1.9 ± 1.5	4.6 ± 2.4	1.00
POD3	0.1 ± 0.5	2.6 ± 1.8	<0.001 *

POD; postoperative day, * significant difference with *p* < 0.05.

- *Minimum VAS and time to minimum VAS.* A statistically significant difference in minimum VAS between groups was observed on POD6 and POD7 (*p* < 0.001 for both) (Table 4). However, there was no statistically significant difference in time to minimum VAS between the esflurbiprofen and placebo groups at any time point from POD4 to POD7 (*p* > 0.05 all) (Table 4).

**Table 4:** Comparison of minimum VAS during a day (minVAS) and time to minimum VAS between groups at different time points.

	Minimum VAS (minVAS)			Time to minimum VAS		
	Esflurbiprofen Group	Placebo Group	*p*-Value	Esflurbiprofen Group	Placebo Group	*p*-Value
	(*n* = 35)	(*n* = 35)		(*n* = 35)	(*n* = 35)	
POD4	0.2 ± 0.4	1.9 ± 0.6	0.30	12.8 ± 2.8	13.2 ± 1.7	0.99
POD5	0.1 ± 0.2	1.5 ± 0.6	0.71	12.2 ± 2.5	12.3 ± 1.6	0.99
POD6	0.0 ± 0.2	1.0 ± 0.6	0.01 *	11.9 ± 2.4	12.1 ± 1.3	0.63
POD7	0.0 ± 0.0	0.7 ± 0.7	<0.001 *	11.6 ± 2.2	11.6 ± 1.2	0.68

POD; postoperative day, *; significant difference with *p* < 0.05.

### 3.3. Knee Function Outcomes

**Figure 4:**
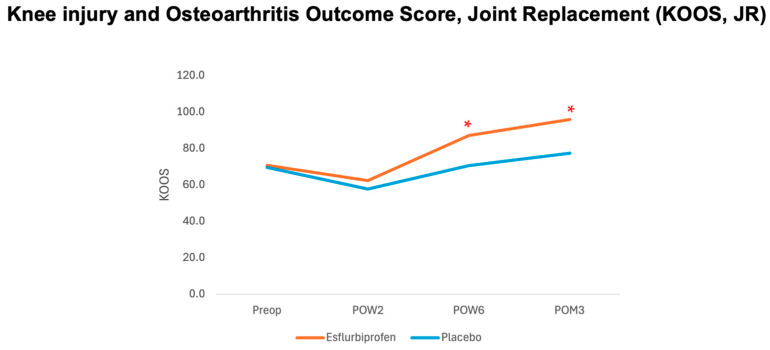
Graph demonstrating relationship between knee injury and osteoarthritis outcome junior score (KOOS, JR) and different time points. Orange and blue lines represent esflurbiprofen and placebo groups, respectively. (* = statistically significance with *p* < 0.05).

- *KOOS, JR score.* Both groups did not show any significant difference in KOOS, JR score on the preoperative period (*p* = 0.607) and POW2 (*p* = 0.339). However, a statistically significant improvement in KOOS, JR scores was found in the Esflurbiprofen group from POW6 to POM3 (*p* < 0.001 for both) compared to the placebo group (Table 5 and Figure 4). At 3-month postoperatively, the mean difference in the KOOS, JR score.

**Table 5:** Comparison of knee injury and osteoarthritis outcome score, joint replacement (KOOS, JR) between groups at different time points.

KOOS, JR	Esflurbiprofen Group	Placebo Group	*p*-Value
	(*n* = 35)	(*n* = 35)	
Preop	72.4 ± 10.7	64.1 ± 12.1	0.60
POW2	59.7 ± 21.2	50.5 ± 20.6	0.34
POW6	88.1 ± 17.4	64.4 ± 20.4	<0.001 *
POM3	96.7 ± 6.5	72.4 ± 14.7	<0.001 *

KOOS, JR; knee injury and osteoarthritis outcome score, joint replacement. POW; postoperative week, POM; postoperative month, * significant difference with *p* < 0.05.

- *Knee flexion angle and knee circumference.* During the 3-month postoperative period, there was no statistically significant difference in knee flexion angle and knee circumference between both groups (*p* > 0.05 for all) (Table 6).

**Table 6:** Comparison of knee flexion angle (degrees) and knee circumference (cm) between groups at different time points.

	Knee Flexion Angle (Degrees)			Knee Circumference (cm)		
	Esflurbiprofen Group	Placebo Group	*p*-Value	Esflurbiprofen Group	Placebo Group	*p*-Value
	(*n* = 35)	(*n* = 35)		(*n* = 35)	(*n* = 35)	
Preop	98.0 ± 10.2	97.7 ± 7.0	0.96	39.3 ± 3.8	40.8 ± 3.0	0.99
POD2	87.5 ± 26.7	91.0 ± 30.3	0.66	39.7 ± 8.9	40.3 ± 3.8	0.60
POD3	95.0 ± 18.1	93.3 ± 23.7	0.45	39.8 ± 8.9	40.5 ± 3.6	0.73
POW2	101.3 ± 11.4	102.5 ± 12.9	0.70	39.4 ± 8.7	40.2 ± 3.8	0.78
POW6	106.5 ± 10.7	105.8 ± 12.7	0.73	48.1 ± 58.6	48.9 ± 64.5	0.23
POM3	110.4 ± 10.1	109.8 ± 11.9	0.78	38.4 ± 10.2	38.8 ± 7.6	0.46

POD: postoperative day, POW: postoperative week, POM: postoperative month.

- *Adverse effects.* In the esflurbiprofen group, one patient (*n* = 1, 2.78%) had experienced an erythematous rash since the first application day and was advised to stop using the patch immediately. The rash was then spontaneously resolved within 24 h after removing the patch. The data from this patient were analyzed with intention-to-treat analysis. Placebo group had no adverse effect (*n* = 0, 0%). Therefore, no statistically significant difference was found between the groups (*p* = 1.00).

## 4. Discussion

This study demonstrated the efficacy of 14-day transdermal NSAIDs patch application after TKA for statistically significant reduction in the min-VAS (on POD6-7), postoperative pain at rest (rVAS) and on movement (mVAS) (from POD7 to POM3), and morphine consumption (on POD3) and for statistically significant improvement of the postoperative KOOS, JR score (from POW6 to POM3) without a significant difference in adverse effect or complication, compared to placebo.

Regards to the postoperative pain outcomes during the perioperative period (POD1-3), our findings showed no significant difference between groups for rVAS and mVAS and a statistically significant reduction in morphine consumption in POD3 (Table 2 and Table 3 and Figure 3). These results were not aligned with the previous study by Tsuji et al. [15], which had statistically significant better pain scores on days 1 and 3 compared to the control group. This could be explained by the difference in patch application method in our study (1 patch per day started from POD3) and the previous study (2 patches per day started immediately after TKA), resulting in the statistically significant reduction in morphine consumption on POD3. Moreover, we hypothesized that the difference in multimodal analgesia protocol used between this study (combined spinal anesthesia and subsartorial block, periarticular injection, and intravenous dexamethasone) and the previous study (general anesthesia and periarticular injection) might play a role. Previous meta-analyses have confirmed the effectiveness of multimodal analgesia techniques as spinal anesthesia and peripheral nerve blocks, periarticular injection, and intravenous dexamethasone [21,22,23]. Combined spinal anesthesia and peripheral nerve blocks, including subsartorial block, are safe and effective for pain control in TKA [21]. Previous meta-analysis study showed that the periarticular injection significantly reduces mVAS at 24 h by a standardized mean difference of 0.53 (95% confidence interval [CI] −0.80 to −0.25) [22]. Also, the added on intravenous dexamethasone in TKA significantly reduce rVAS, mVAS, and morphine consumption at 24 and 48 h (rVAS at 24 and 48 h: −0.71 [95% CI −0.86 to −0.55] and −0.30 [95% CI −0.43 to −0.18], mVAS at 24 and 48 h: −0.89 [95% CI −1.23 to −0.55] and −0.42 [95% CI −0.62 to −0.23], and morphine consumption at 24 and 48 h (−3.06 [95% CI −4.82 to −1.30] and −5.23 [95% CI −8.28 to −2.18] [23]. Due to the effectiveness of multimodal analgesia in this study, our patients would experience less postoperative pain and resulting in easier pain control and enhancing rehabilitation after hospital discharge.

After hospital discharge, our findings showed a statistically significant reduction in minVAS on POD6-7 (Table 4), both rVAS and mVAS from POD7 to POM3 (Figure 3 and Table 2). Post hoc power analysis for mVAS on POD7 was 100%. These findings were also not comparable with the previous study by Tsuji et al., which had no significant difference in VAS scores on days 5, 7, and 14 [15]. This could be explained by the effect of Esflurbiprofen patch application and the effectiveness of early postoperative pain management by multimodal analgesia and the IA-TXA application in this study. Esflurbiprofen patch application for 14 days significantly reduces the postoperative pain and controls the inflammation during the time of application (from POD3 to POW2). Moreover, a previous study included a demonstration that the IA-TXA injection in TKA significantly reduces the inflammation markers (fibrinogen, erythrocyte sedimentation rate, C-reactive protein, interleukin-6) on POD3 [24]. When combined with the effective multimodal analgesia protocol, the postoperative pain would be controlled more easily by postoperative medications and physiotherapy, resulting in the continuous improvement in postoperative pain after stopping the patch application (from POW6 to POM3) (Table 2 and Figure 3).

In terms of postoperative knee function, knee circumference, and knee flexion angle, the findings in the present study revealed that the postoperative KOOS, JR score statistically significantly improved with the 14-day esflurbiprofen patch application on POW6 and POM3 without a significant difference in knee circumference and knee flexion angle compared to the control group. This could be explained by the correlation between the effect of postoperative knee functional recovery and the postoperative pain control in our study, especially on the mVAS. We hypothesized that the daily application of esflurbiprofen patch during a 2-week postoperative period after TKA would affect the postoperative inflammation around the knee joint, which would reduce both pain at rest and pain upon movement, resulting in better physical function in daily activities and improving knee function. However, this study did not show any significant difference in knee circumference and knee flexion angle between the two groups. The results were inconsistent with those in the previous study by Tsuji et al., in which they demonstrated a significant increase in knee flexion angle on postoperative days 7 and 14 [15]. This could be explained by the dosage used in this study (1 patch per day) compared to the previous study (2 patches per day) and the effect of IA-TXA injection. In addition to the effect of IA-TXA for reducing postoperative inflammation, a previous study by Pandher DS et al. showed that the IA-TXA injection resulted in a better range of motion at 48 h after TKA [25]. Therefore, a combined esflurbiprofen patch and IA-TXA would result in superior postoperative pain control and facilitate early knee rehabilitation after TKA.

Regards to the clinical significance of postoperative pain reduction and functional outcome improvement in this study, previous studies showed that the minimal clinical important difference (MCID) for VAS and 3-month KOOS, JR were 1.8 and 16.9, respectively [20,26]. Therefore, the findings from the present study have proven that the Esflurbiprofen patch application clinically significantly reduces mVAS at POW6 and POM3 and KOOS, JR at POM3 (Figure 3 and Figure 4, Table 2 and Table 5).

The strengths of our study are the well-designed triple-blinded randomized controlled trial, completed follow-up, and the intention-to-treat analysis. On the other hand, some limitations should be considered. First, although both groups were blinded to the group allocation by using a placebo patch, the expectation of patch therapy might be a possible confounder in our study. Moreover, the application of an NSAID patch on POD3 was designed due to the standard perioperative management after TKA in our institution, as using a drain and compressive dressings for 2 days (from POD0 to POD2). Therefore, the maximal analgesic effect of the NSAIDs patch might be potentially underestimated, and the optimum dosage or duration of the transdermal esflurbiprofen patch has not been explored. Second, the oral NSAIDs were not considered due to their systemic adverse effects, such as gastrointestinal bleeding or renal failure. Notwithstanding, Miyake et al. reported that the 2-week topical esflurbiprofen patch application after total hip arthroplasty provided comparable analgesic effects to 2-week oral celecoxib. In addition, the former did not have any systemic or local adverse effects, while the latter came up with 4% drug-induced renal dysfunction and 2% gastrointestinal disturbance [7]. Lastly, the follow-up time in this study was only 3 months, so long-term safety and efficacy of NSAIDs patch application after TKA are still unknown. Nevertheless, A previous study by Yataba et al. showed that topical esflurbiprofen could be applied up to 52 weeks without serious adverse effects in patients with knee OA [11]. Therefore, future studies on topical NSAIDs patch application were recommended for evaluation of the effectiveness and safety of this method among postoperative TKA patients.

## 5. Conclusions

In conclusion, the 2-week application of an esflurbiprofen patch after TKA demonstrated significant advantages in reducing both postoperative pain at rest and on movement, reducing morphine consumption, and improving the postoperative knee functional outcomes in the early postoperative period up to 3 months. This substantial reduction in postoperative pain after 2 weeks highlights its potential role in enhancing postoperative functional recovery after TKA. Future studies should explore its impact and clinical utility in postoperative pain management.

## Figures and Tables

**Table 1:** Demographic data.

	Esflurbiprofen Group	Placebo Group	*p*-Value
	(*n* = 35)	(*n* = 35)	
Age, year ^a^	69.3 ± 8.5	69.9 ± 6.0	0.77
BMI, kg/m^2 a^	26.7 ± 4.3	26.9 ± 4.7	0.89
Male:female ^b^	8:27	4:31	0.34
Left:right ^b^	19:16	18:17	1.00
Comorbid disease ^c^			
Hypertension	28 (80.0%)	28 (80.0%)	1.00
Diabetes	6 (17.1%)	10 (28.6%)	0.37
Ischemic heart disease	5 (14.3%)	6 (17.1%)	0.48
Chronic kidney disease	3 (8.6%)	1 (2.9%)	1.00
Stroke	4 (11.4%)	5 (14.3%)	0.48
Implant used ^c^			
NexGen	8 (22.9%)	7 (20.0%)	0.20
Attune	13 (37.1%)	7 (20.0%)	
PFC Sigma	14 (40.0%)	21 (60.0%)	

^a^; value presented as mean ± standard deviation, ^b^; value presented as ratio, ^c^; value presented as number of cases (percentage).

## Data Availability

The data presented in this study are available on request from the corresponding author due to participants’ privacy concerns.

## Abbreviations

The following abbreviations are used in this manuscript:

TKA	Total Knee Arthroplasty
VAS	Visual Analog Scale
KOOS, JR	Knee Osteoarthritis Outcome Score, Joint Replacement
OA	Osteoarthritis
NSAIDs	Non-steroidal anti-inflammatory drugs
IA-TXA	Intraarticular tranexamic acid
POD	Postoperative day
POW	Postoperative week
POM	Postoperative month
BMI	Body Mass Index
rVAS	VAS during the rest period
mVAS	VAS during the movement
Min-VAS	Minimum VAS
